# Compressive cryotherapy versus cryotherapy alone in patients undergoing knee surgery: a meta-analysis

**DOI:** 10.1186/s40064-016-2690-7

**Published:** 2016-07-13

**Authors:** Mingzhi Song, Xiaohong Sun, Xiliang Tian, Xianbin Zhang, Tieying Shi, Ran Sun, Wei Dai

**Affiliations:** Department of Orthopaedics, The First Affiliated Hospital of Dalian Medical University, 222 Zhong Shan Road, Dalian, 116011 Liaoning People’s Republic of China; Department of Orthopaedics, The Third Affiliated Hospital of Dalian Medical University, 378 Shi Ji West Road, Jinpu New Area, 116200 Liaoning People’s Republic of China; Department of Nursing, The First Affiliated Hospital of Dalian Medical University, 222 Zhong Shan Road, Dalian, 116011 Liaoning People’s Republic of China; Department of Hepatobiliary Surgery, The First Affiliated Hospital of Dalian Medical University, 222 Zhong Shan Road, Dalian, 116011 Liaoning People’s Republic of China; Operation Room, The First Affiliated Hospital of Dalian Medical University, 222 Zhong Shan Road, Dalian, 116011 Liaoning People’s Republic of China

**Keywords:** Cryotherapy, Meta-analysis, Nursing, Surgery, Total knee arthroplasty, Anterior cruciate ligament reconstruction, Arthroscopy, Postoperative care, Postoperative complications, Pain, Edema, Pain relief

## Abstract

**Aim:**

This study aims to conduct a meta-analysis to identify and compare the effectiveness of compressive cryotherapy and cryotherapy alone for patients undergoing knee surgery.

**Background:**

Postoperative management is an important guarantee for the success of surgery. Cryotherapy and compression are two common nursing techniques after knee surgery, and are considered to be effective for postoperative clinical symptoms such as local pain and swelling. However, no previous meta-analyses have compared the effectiveness of compressive cryotherapy and cryotherapy alone in patients undergoing knee surgery.

**Design:**

A meta-analysis of randomized controlled trials (RCTs).

**Methods:**

We conducted a search in MEDLINE (via Pubmed, 1990–2014), EMBASE (via Elsevier, 1990–2014), Cochrane Central Register of Controlled Trials (The Cochrane Library, 1990–2014), CINAHL (1990–2014) and China National Knowledge Infrastructure (1990–2014) databases for RCTs published in English and Chinese. The primary outcome measure of interest was visual analog scale and girth measure. Finally, a meta-analysis was carried out using RevMan 5.3.

**Results:**

Among the 593 RCTs, 10 RCTs were selected and included into this study. These studies included 522 patients who underwent knee surgery. Patients who underwent compressive cryotherapy tended to have less pain than patients who underwent cryotherapy alone at POD2 and POD3, while compressive cryotherapy had a strong tendency towards less swelling over cryotherapy alone at POD1 and POD2. However, there was no significant difference between compressive cryotherapy and cryotherapy alone at the intermediate stage of rehabilitation after knee surgery. All adverse reactions were recorded in all included RCTs.

**Conclusion:**

Current evidence suggests that compressive cryotherapy is beneficial to patients undergoing knee surgery at the early rehabilitation stage. At the last stage, the effectiveness of compressive cryotherapy and cryotherapy alone were found to be similar.

## Background

 Total knee arthroplasty (TKA), knee arthroplasty, anterior cruciate ligament (ACL) reconstruction and arthroscopic therapy have become the most common knee surgical methods. Numerous patients suffering from knee diseases have achieved satisfactory curative effects through these methods. However, severe clinical symptoms including local pain, swelling and reduced knee range of motion have frequently occurred during the postoperative period (van Grinsven et al. 2010; Yabroudi and Irrgang 2013). They were generally treated as main factors that delay functional recovery time. Therefore, effective postoperative management appears to be particularly important for the operative success that surgeons have contributed.

Cryotherapy or cold treatment is the traditional treatment, which is affordable, easy to perform and widely applied for acute musculoskeletal injuries. Cold has extensive roles in tissue injury recovery, which mainly include reducing cellular metabolism, delaying nerve conduction, inhibiting edema expansion and alleviating pain (Nadler et al. 2004; Warren et al. 2004; Cohn et al. 1989). To some extent, surgery was been regarded as an acute injury. Therefore, cryotherapy has been gradually applied in postoperative patients. In addition to relieving pain and edema, cryotherapy is also helpful in promoting healing; enabling patients to return to their regular activities. Several researchers have achieved these exact results, which were obtained through comparative studies that evaluated the effectiveness of cryotherapy in patients after knee surgeries (Cohn et al. 1989; Lessard et al. 1997). Evidence-based medicine studies have indicated that patients undergoing TKA and arthroscopic ACL reconstruction have benefited from postoperative cryotherapy (Ni et al. 2015; Adie et al. 2010; Martimbianco et al. 2014). Since nurses have begun to be involved in decision-making, cryotherapy has become an important postoperative management. Through thousands of years of development and evolvement, cryotherapy could now be applied through different methods such as cold dressing, cold packs, crushed ice bags, cooling pads and cold compression devices (CCD). Due to the facilitation of the operation process, more nurses have continued to select CCD to perform cryotherapy. In fact, compared with traditional cryotherapy, CCD simultaneously brings compression into postoperative management. Several clinical studies on the effectiveness of compression have been performed without inconclusive results, and compression remains to be seen as a common postoperative intervention (Charalambides et al. 2005; Smith et al. 2002; Andersen et al. 2008; Pinsornsak and Chumchuen 2013; Cheung et al. 2014; Munk et al. 2013). To date, compressive cryotherapy (CC) has been shown to significantly reduce postoperative pain scores after TKA, ACL reconstruction and wrist arthroscopy (Meyer-Marcotty et al. 2011; Markert 2011; Schröder and Pässler 1994). Although cryotherapy has exhibited a clear effect in promoting recovery for postoperative patients, few studies have compared CC and cryotherapy alone (CA). For guidance in postoperative nursing, this kind of comparison has become more significant. The viewpoint on this comparison is very inconsistent in academia at present. The study of Kraeutler et al. indicated that CC by CCD did not reduce postoperative pain in patients undergoing shoulder arthroscopy (Kraeutler et al. 2015). However, the opposite view for CC has also attracted the people’s attention (Schröder and Pässler 1994). Fortunately, studies that have compared CC and CA in patients after knee surgery are relatively enough to attain a conclusion. Since randomized controlled trials (RCTs) have been published over the past 24 years, we decided to critically appraise and synthesize existing evidences obtained when effectiveness was compared between compressive cryotherapy and cryotherapy alone following knee surgery. This review would help explore the impact of compression on cryotherapy, assist in clinical and nursing decisions for selecting the optimum cryotherapeutic method, and study the gaps in this area.

## Aim and methods

This study conducted a meta-analysis to identify and compare the effectiveness of CC and CA in patients undergoing knee surgery. For this study, the review process of the Cochrane Collaboration was adopted, including the identification of a priori inclusion/exclusion criteria (Higgins and Green 2011). Furthermore, the review included the development of a structured clinical question linked to the comprehensive and detailed search of literature using appropriate databases and a priori inclusion and exclusion criteria, the systematic extraction and recording of study characteristics, methods, findings and methodological qualities, and the synthesis of comparable studies. Two independent reviewers were involved in each stage, and their extractions and appraisals were cross-referenced to ensure accuracy.

### Eligibility criteria

Randomized controlled trials were selected for this study. Skeletally mature patients (18 years old) were submitted to primary TKA, knee arthroplasty, ACL reconstruction and arthroscopic surgery. Studies that included patients with bilateral and secondary surgery were excluded. Any type of compressive cryotherapy around the knee (e.g. CCD and home-made equipment) compared to cryotherapy alone around the knee (e.g. cold dressing, cold packs, cooling pads, crushed ice bags and CCD) were interventions that needed to be studied. CCD is a compressive cooling system [e.g. Cryocuff system (Aircast Cryocuff, Inc., Summit, New Jersey, USA), Game ready (CoolSystems, Inc., Alameda, California, USA) coolsystems and Ever-cryo system (Cryo-Push Medical Technology Co., Ltd., Chengdu, Sichuan, China)] that can supply a controlled cryogenic circulation and generate focal compression to the knee.

### Outcome measures

(1) Pain intensity [e.g. measured by visual analog scale (VAS)] (2) swelling (e.g. knee circumference measured using tape), and (3) adverse events (thermal injury such as frostbite and transient nerve palsy).

### Search strategy

The following databases were searched: MEDLINE (via Pubmed, 1990–2014); EMBASE (via Elsevier, 1990–2014); Cochrane Central Register of Controlled Trials (The Cochrane Library, 1990–2014); CINAHL (1990–2014), and China National Knowledge Infrastructure (1990–2014). ClinicalTrials.gov was also searched for ongoing and recently completed trials. Studies that were published in English and Chinese were included into this study. The search was complemented by screening the reference lists of retrieved articles. These search strategies were based on the strategy developed for MEDLINE (via Pubmed), combined with the high-pass sensitivity filter developed by the Cochrane Collaboration, to identify RCTs (Higgins and Green 2011). The following search terms were used: “Anterior Cruciate Ligament” OR “Anterior Cruciate Ligament Reconstruction” OR “Arthroplasty, Replacement, Knee” OR “Arthroscopy”, AND “Cryotherapy”; and related terms adapted for each database. In addition, in order to search for relevant studies, experts in the field were consulted; and the reference list of all these studies were individually checked for additional studies. Studies published prior to 1990 were excluded due to changes in technology and patient care and populations.

### Search outcome

**Fig. 1:**
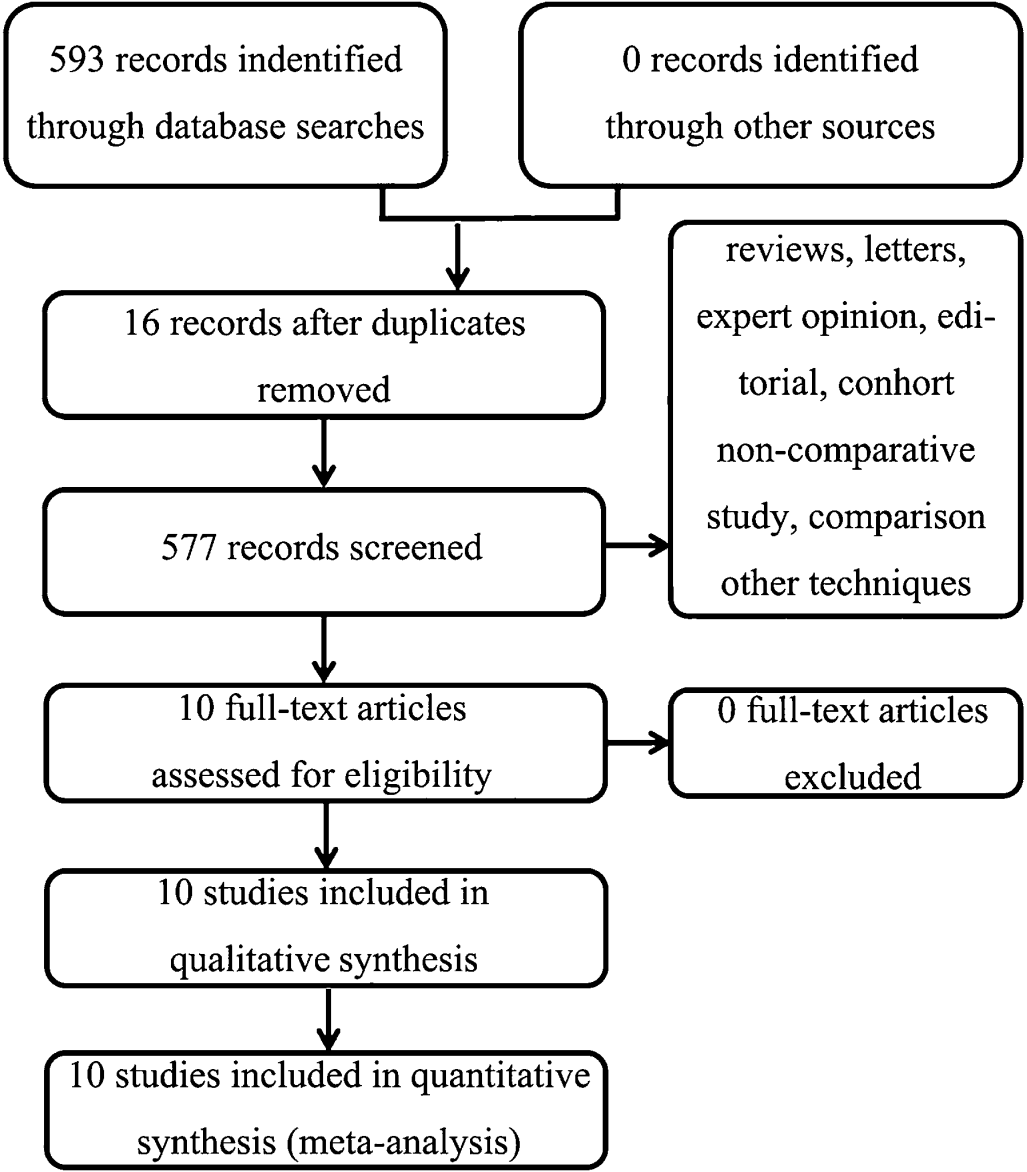
Study selection process

The initial search identified 593 possible studies. The screening of titles, abstracts and full papers against the inclusion criteria resulted in the selection of ten studies (Fig. 1). Studies with postoperative recovery (including VAS and/or girth measure) records were included in the pooling.

### Quality appraisal

**Fig. 2:**
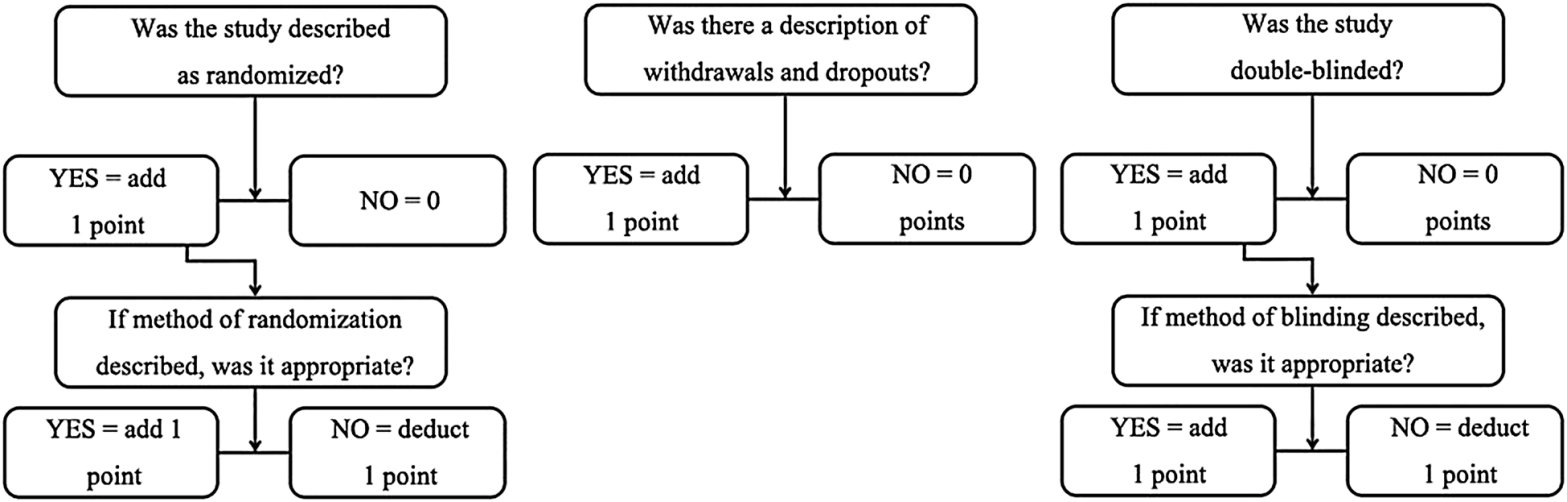
JADAD scale

The methodological quality of these trials was assessed through the JADAD scale, a tool for assessing the quality of RCTs, through the evaluation of blinding, randomization and losses reported (Jadad et al. 1996) (Fig. 2). Additionally, bias in treatment intention, prognosis characteristics, regional differences, amount of losses and follow-up was assessed; but these were not used as exclusion criteria. The full texts of studies that were considered potentially relevant were obtained and read independently by the same two reviewers. Studies that fulfilled the aforementioned selection criteria were included in the meta-analysis. Disagreements between two reviewers were decided by a third reviewer.

### Data extraction

Two independent reviewers (TXL and ZXB) extracted data from all included studies using a standardized extraction form especially created for this meta-analysis. The form contained information of the participants, the methodological aspects of the study, interventions, and measured outcomes. These two individual forms were discussed by the reviewers until a consensus was reached, and these forms were merged into a single extraction form. Persistent disagreements were settled by a third reviewer. When necessary, the authors of these included studies were contacted for further information.

### Quantitative data synthesis and analysis

For the meta-analysis component, raw unstandardized mean differences of VAS and postoperative girth measure (at the joint line level of the operative knee) at postoperative day (POD) 1–3, as well as at postoperative week (POW) 1 and 2, were extracted from studies that provided these data. This pooling method was selected, because this measure (mean VAS and girth measure) is meaningful for evaluating therapeutic efficacy, well-recognized as valid, and the most common form of measurement across similar studies. VAS scoring with a range of 0–10 or 0–100 was used to evaluate subjective pain intensity. Furthermore, VAS scores were used to assess the postoperative pain condition of patients who received CC and CA in all included studies. VAS scores at POD1, POD2, POD3, as well as at POW1 and POW2, were included into the meta-analysis. Girth measure at mid-patella level was commonly performed to evaluate the postoperative swelling condition of patients. For this study, the value of swelling equals the circumference of the operative knee minus the circumference of the healthy knee. Finally, swelling at POD1, POD2 and POD3 were included into the meta-analysis. Due to the likelihood of diversity between the selected trials, settings and populations, a random effects model was used to synthesize these data. The standardized mean difference (SMD) was calculated to allow for two different VAS scales (Lakhan et al. 2015). In addition, heterogeneity was considered to be low when I_2_ was ≤25 %, moderate when I_2_ was ≤50 %, high when I_2_ was ≤75 %, and very high when I_2_ was >75 % (Higgins and Green 2011). All calculations were performed via the RevMan 5 software (Review Manager 5.3.5, Cochrane Collaboration). *P* values were considered statistically significant at ≤0.05.

## Results

### Literature search

A total of 593 potential trials were identified via the first search strategy. Then, 577 reports were excluded during the screening of titles, and six reports were excluded after screening of abstracts. Finally, 583 reports were excluded according to eligibility criteria. No additional studies were obtained after the reference review. After careful full-text evaluation, ten independent RCTs (Schröder and Pässler 1994; Li et al. 2010a, b; Demoulin et al. 2012; Waterman et al. 2012; Tian et al. 2013; Xie et al. 2013, 2014; Xu et al. 2013; Wang et al. 2014) with 522 patients were included in the current meta-analysis.

### Study characteristics

The main characteristics of the included studies are listed in Table 1. The sample size of the included studies ranged from 32 to 140 patients. The methods used for surgery were mostly arthroscopic surgery (*n* = 7 trials), ACL reconstruction (*n* = 3 trials) and TKA (*n* = 2 trial). Statistically similar baseline characteristics were observed between the CC and CA groups, including age and gender. The frequency, number and implementation methods of compression and cryotherapy varied among studies. However, methods used for assessing postoperative pain and swelling were VAS and girth measure, respectively.

**Table 1:** Main characteristics and findings of ten studies comparing compressive cryotherapy with cryotherapy alone after a knee surgery

Authors	Study design	JADAD score	Participants	Intervention	Outcomes measured	Results
Schröder and Pässler (1994)	RCT	1	44 Patients (all underwent ACL reconstruction surgery under arthroscopy) G1 (n = 21) 15 M/6 W Mean age 24.2 y G2 (n = 23) 18 M/5 W Mean age 24.8 y	G1: CCD (continuously up to hospital discharge) G2: Ice bags (three times/day)	1. Pan intensity (VAS) 2. Edema (knee circumference measured using tape) 3. Range of motion-ROM (in degrees) 4. Knee function (knee score of Noyes and McGinniss) 5. Use of analgesic medication (total doses, mg/kg) of oral tilidine, IM pethidine and piritramide 6. Blood loss (in ml) 7. Adverse events Outcomes were measured on POD 1, 2, 3, 6, 14, 28	G1 had significant differences in: ROM on all days (P < 0.01); VAS pain scale on the 6th day (P < 0.01); knee edema on the 3rd and 6th days (P < 0.035), knee function (P < 0.025) and used less oral tilidine and IM piritramide (P < 0.04) There were no adverse events
Li et al. (2010a)	RCT	3	140 Patients (all underwent arthroscopic surgery) G1 (n = 70) G2 (n = 70) Total mean age 36.6 y	G1: CCD (continuously up to POD2) G2: Conventional ice pack therapy (cycles (1 h treatment and 1 h pause) for 1 day)	1. Pain intensity (VAS) 2. Use of analgesic medication (celecoxib)	G1 had significantly lower VAS pain scores and less use of pain medications on 6, 12, 24, 36, 48 h after operation (P < 0.05) There were no adverse events
Li et al. (2010b)	RCT	3	140 Patients (all underwent arthroscopic surgery) G1 (n = 70) G2 (n = 70) Total mean age 36.6 y	G1: CCD (continuously up to POD2) G2: Conventional ice pack therapy (cycles (1 h treatment and 1 h pause) for 1 day)	Edema (knee circumference measured using tape)	G1 had significantly lower VAS pain scores and less use of pain medications on 6, 12, 24, 36 and 48 h after operation (P < 0.05) There were no adverse events
Demoulin et al. (2012)	RCT	3	66 Patients (all underwent primary unilateral TKA surgery) G1 (n = 22) 8 M/14 W Mean age 72.0 y G2 (n = 22) 9 M/13 W Mean age 68.1 y G3 (n = 22) 9 M/13 W Mean age 71.2 y	G1: GCD (3 sessions (90 s)/day from POD2 to hospital discharge) G2: Traditional ‘‘gel pack’’ therapy (5 sessions (20 min)/day from POD2 to hospital discharge) G3: CCD (5 sessions (20 min)/day from POD2 to hospital discharge)	1. Pain intensity (VAS) 2. Edema (knee circumference measured using tape) 3. Range of motion-ROM (in degrees) 4. Cutaneous temperature	Comparison including VAS, edema, cutaneous temperature and ROM between G2 and G3 on POD7 remained non-significant (P > 0.05). There were no adverse events
Waterman et al. (2012)	RCT	3	36 Patients (all underwent ACL reconstruction surgery) G1 (n = 18) 15 M/3 W Mean age 28.7 y G2 (n = 18) 15 M/3 W Mean age 30.9 y	G1: CCD (3 sessions (30 min)/day for 6 weeks) G2: Conventional ice pack therapy (3 sessions (30 min)/day for 6 weeks)	1. Pain intensity (VAS) 2. Edema (knee circumference measured using tape) 3. Use of analgesic medication (not reported) 4. Knee function (Lysholm score) 5. Quality of life (SF-36) Outcomes were measured on POW 1, 2, and 6	G1 had significantly lower VAS pain scores (P < 0.0001) and discontinued use of pain medications, by 6 weeks (P = 0.0008) There were no adverse events
Tian et al. (2013)	RCT	3	64 Patients (all underwent arthroscopy surgery) G1 (n = 32) 7 M/25 W Mean age 54.21 y G2 (n = 32) 12 M/20 W Mean age 56.44 y	G1: CCD (continuous treatment for 2 days after operation) G2: Conventional ice pack therapy (cycles (30 min treatment and 8 h pause) for 2 days after operation)	1. Pain intensity (VAS) 2. Edema (knee circumference measured using tape) 3. Knee function (HSS score) 4. Comfort degree	G1 had significantly lower VAS pain scores and lighter edema on POD1 and 2 (P < 0.05) G1 had higher comfort degree than G2 (P < 0.05). There were no adverse events
Xie et al. (2013)	RCT	1	40 Patients (all underwent ACL reconstruction surgery under arthroscopy) G1 (n = 20) 16 M/4 W Mean age 29.1 y G2 (n = 20) 15 M/5 W Mean age 28.2 y	G1: CCD (continuous treatment for 3 days after operation) G2: Physiological saline ice pack therapy (4 sessions (45 min)/day for 3 days after operation)	1. Pain intensity (VAS) 2. Edema (knee circumference measured using tape)	G1 had significantly lower VAS pain scores and lighter edema on POD1, 2 and 3 (P < 0.05) There were no adverse events
Xu et al. (2013)	RCT	3	60 Patients (all underwent TKA surgery) G1 (n = 30) 11 M/19 W Mean age 65.8 y G2 (n = 30) 10 M/20 W Mean age 67.1 y	G1: CCD (continuous treatment for 2 days after operation) G2: Hypertonic saline crushed ice bag (cycles (20 min treatment and 2 h pause) for 3 days after operation)	1. Pain intensity (VAS) 2. Edema (knee circumference measured using tape) 3. Range of motion-ROM (in degrees) 4. Postoperative drainage 5. Cutaneous temperature	G1 had significantly lower VAS pain scores and lighter edema on POD1 and 2 (P < 0.05) G1 had better ROM on POD1, 2 and 3 (P < 0.05) Cutaneous temperature in G1 was higher than G2 (P < 0.05) There were no adverse events
Xie et al. (2014)	RCT	1	40 Patients (all underwent non-ACL reconstruction surgery under arthroscopy) G1 (n = 20) 16 M/4 W Mean age 36.8 y G2 (n = 20) 17 M/3 W Mean age 35.5 y	G1: CCD (continuous treatment for 2 days after operation) G2: Physiological saline ice pack therapy (4 sessions (45 min)/day for 2 days after operation)	1. Pain intensity (VAS) 2. Edema (knee circumference measured using tape) 3. Range of motion-ROM (in degrees)	G1 had significantly lower VAS pain scores and lighter edema on POD1 and 2 (P < 0.05) G1 had higher ROM than G2 (P < 0.05). There were no adverse events
Wang et al. (2014)	RCT	3	32 Patients (20 patients underwent ACL reconstruction surgery and 12 patients underwent PCL reconstruction surgery under arthroscopy) G1 (n = 16) 4 M/12 W Mean age 50.72 y G2 (n = 16) 7 M/9 W Mean age 57.13 y	G1: CCD (continuous treatment for 2 days after operation) G2: Conventional ice pack therapy for 2 days after operation)	1. Pain intensity (VAS) 2. Edema (knee circumference measured using tape) 3. Knee function (Lysholm score) 4. Comfort degree	G1 had significantly lower VAS pain scores and slighter edema on POD1 and 2 (P < 0.05) G1 had higher comfort degree than G2 (P < 0.05). There were no adverse events

*G* group, *M* male, *F* female, *y* years-old

### Risk of bias assessment

Based on the JADAD scale, the maximum score among these included studies (Jadad et al. 1996) was 3; because it is not possible to have a double-blind study in this field. Overall methodological quality was moderate (7 trials, JADAD Score = 3), while three trials were of moderate to low quality (JADAD Score = 1). It was not possible to test for publication bias due to the number of trials, in which the outcomes that could be synthesized was too small.

### Meta-analysis outcomes

#### VAS scores at POD1

**Fig. 3:**
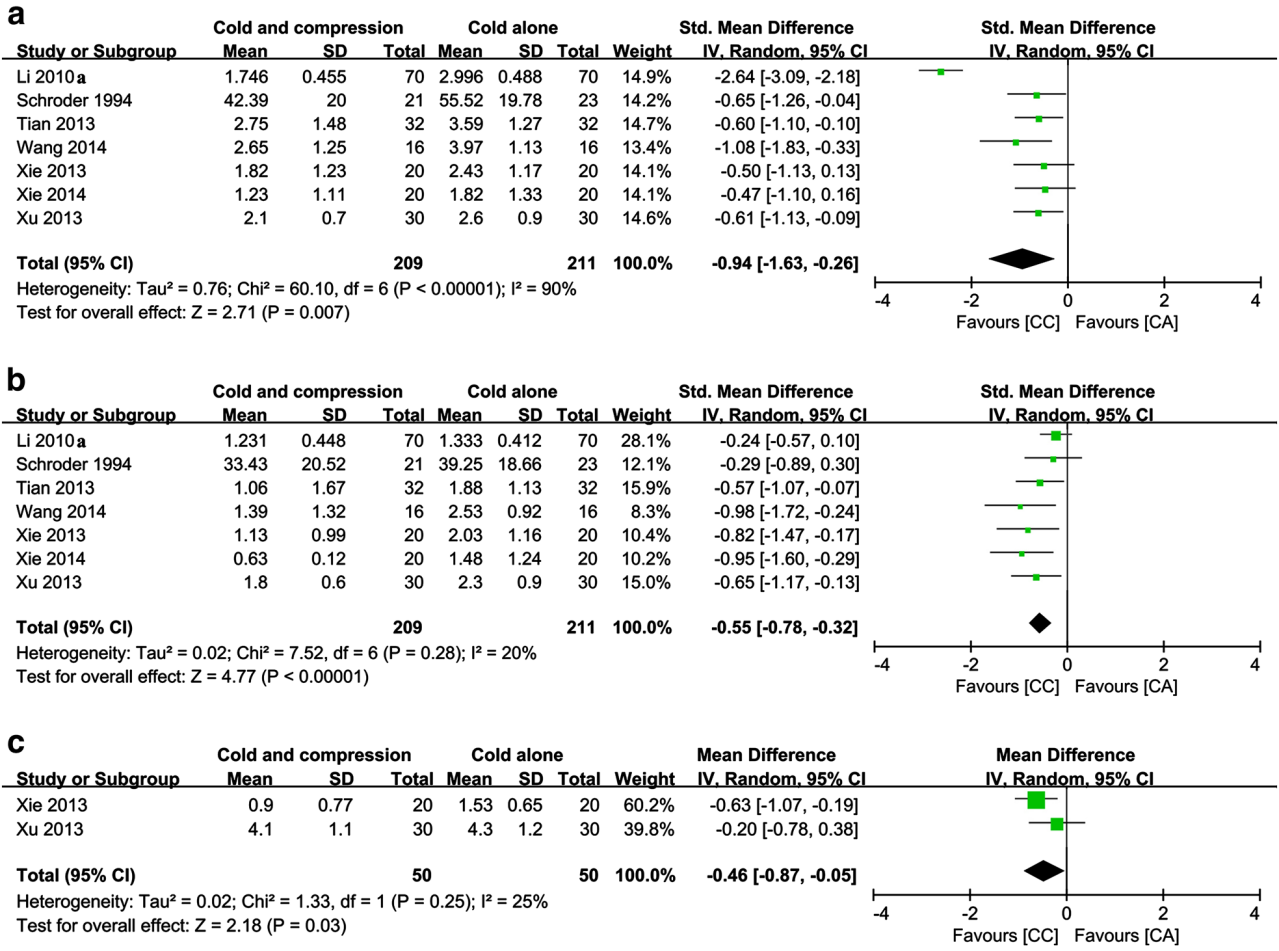
Pooled data of VAS for compressive cryotherapy versus cryotherapy alone at the early rehabilitation stage: **a**–**c** stand for VAS at POD 1, 2 and 3, respectively

Seven included studies included VAS scores at POD1 (Schröder and Pässler 1994; Li et al. 2010a; Tian et al. 2013; Xie et al. 2013, 2014; Xu et al. 2013; Wang et al. 2014). Although these pooled results have indicated that the remission effect of CC on postoperative pain was better than that of CA [MD (mean difference) = −0.94, 95 % CI −1.63 to −0.26, *P* = 0.007], there was significant heterogeneity (Chi^2^ = 60.10, df = 6, *I*^*2*^ = 90 %, *P* < 0.00001; Fig. 3a).

#### VAS scores at POD2

Seven studies reported VAS scores at POD2 (Schröder and Pässler 1994; Li et al. 2010a; Tian et al. 2013; Xie et al. 2013, 2014; Xu et al. 2013; Wang et al. 2014), and there was no significant heterogeneity (Chi^2^ = 7.52, df = 6, *I*^*2*^ = 20 %, *P* = 0.28; Fig. 3b). Pooled results indicated that the remission effect of CC was better than that of CA, and there was a statistically significant difference between these two groups (MD = −0.55, 95 % CI −0.78 to −0.32, *P* < 0.00001).

#### VAS scores at POD3

Two included studies assessed VAS scores at POD3 (Xie et al. 2013; Xu et al. 2013), and there was no significant heterogeneity (Chi^2^ = 1.33, df = 1, *I*^*2*^ = 25 %, *P* = 0.25; Fig. 3c). Pooled results indicate that the remission effect of CC was better than that of CA, and there was a statistically significant difference between these two groups (MD = −0.46, 95 % CI −0.78 to 0.38, *P* = 0.03).

#### VAS scores at POW1

**Fig. 4:**
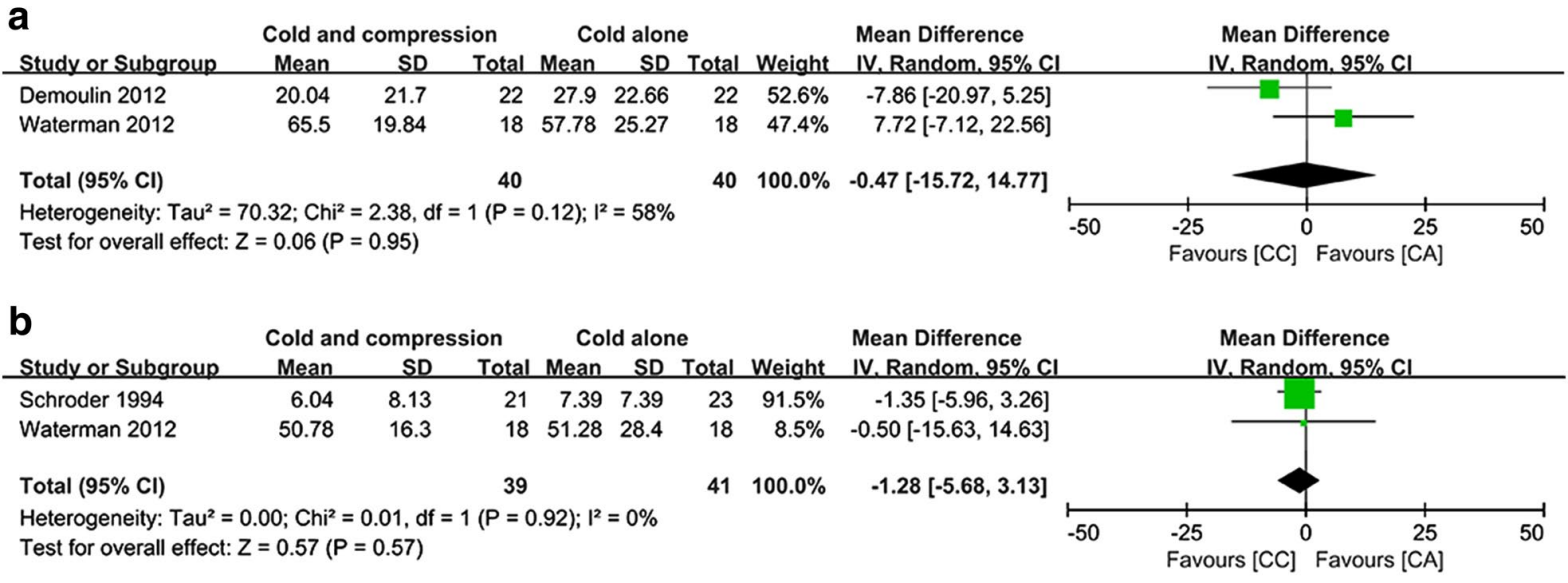
Pooled data of VAS for compressive cryotherapy versus cryotherapy alone at the chronic rehabilitation stage: **a**, **b** stand for VAS at POW 1 and 2, respectively

Two included studies assessed VAS scores at POW1 (Demoulin et al. 2012; Waterman et al. 2012), and there was significant heterogeneity (Chi^2^ = 2.38, df = 1, *I*^*2*^ = 58 %, *P* = 0.12; Fig. 4a). Pooled results indicated that there was no significant difference in pain remission effect between these two groups (MD = −0.47, 95 % CI −15.72 to 14.77, *P* = 0.95).

#### VAS scores at POW2

Two included studies evaluated VAS scores at POW2 (Schröder and Pässler 1994; Waterman et al. 2012), and there was no significant heterogeneity (Chi^2^ = 0.01, df = 1, *I*^*2*^ = 0 %, *P* = 0.92; Fig. 4b). Pooled results revealed that there was no significant difference in pain remission effect between these two groups (MD = −1.28, 95 % CI −5.68 to 3.13, *P* = 0.57).

#### Girth measurement at POD1

**Fig. 5:**
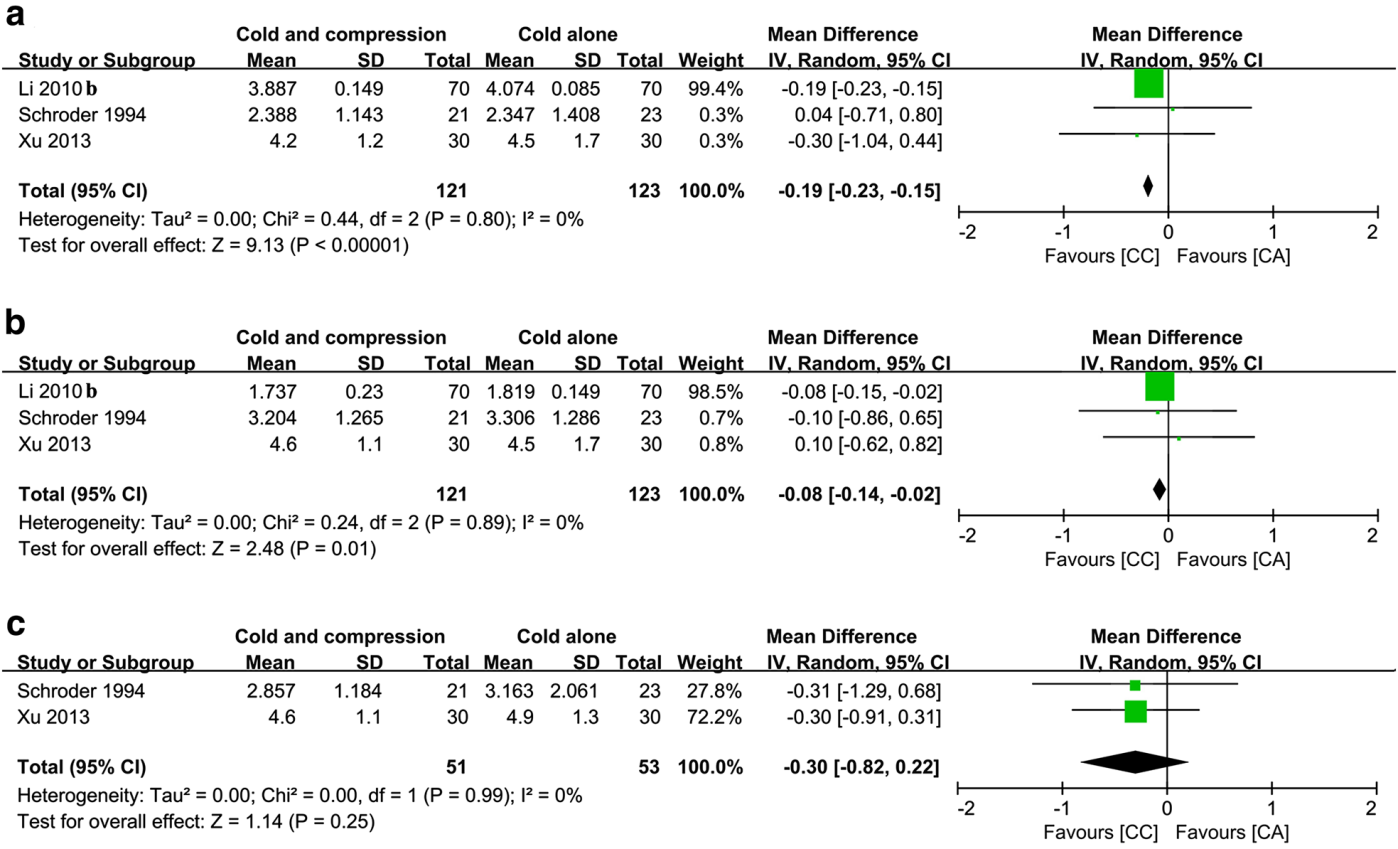
Pooled data of girth measure for compressive cryotherapy versus cryotherapy alone at the early rehabilitation stage: **a**–**c** stand for swelling at POD 1, 2 and 3, respectively

Three studies reported girth measurements at POD1 (Schröder and Pässler 1994; Li et al. 2010b; Xu et al. 2013), and there was no significant heterogeneity (Chi^2^ = 0.44, df = 2, *I*^*2*^ = 0 %, *P* = 0.80; Fig. 5a). Pooled results indicated that swelling in CC was smaller than in CA, and there was a statistically significant difference between these two groups (MD = −0.19, 95 % CI −0.23 to −0.15, *P* < 0.00001).

#### Girth measurement at POD2

Three studies reported girth measurements at POD2 (Schröder and Pässler 1994; Li et al. 2010b; Xu et al. 2013), and there was no significant heterogeneity (Chi^2^ = 0.24, df = 2, *I*^*2*^ = 0 %, *P* = 0.89; Fig. 5b). Pooled results indicated that swelling in CC was smaller than in CA, and there was a statistically significant difference between these two groups (MD = −0.08, 95 % CI −0.14 to −0.02, *P* = 0.01).

#### Girth measurement at POD3

Two studies reported girth measurement at POD2 (Schröder and Pässler 1994; Xu et al. 2013), and there was no significant heterogeneity (Chi^2^ = 0.00, df = 1, *I*^*2*^ = 0 %, *P* = 0.99; Fig. 5c). Pooled results revealed that there was no significant difference in swelling between these two groups (MD = −0.30, 95 % CI −0.82 to −0.22, *P* < 0.25).

#### Adverse reactions

No postoperative adverse reactions occurred in all included studies.

## Discussion

Postoperative satisfaction and functional recovery of patients determine the overall efficacy of knee surgery. Consequently, the methods of postoperative management have achieved a rapid advance. Among these methods, supplying a low cost, convenient and satisfactorily effective cryotherapy has been widely recognized and adopted by both clinical and nursing staffs.

Current clinical studies have demonstrated that cryotherapy after knee surgery may significantly bring immediate benefits by decreasing pain and edema during inflammatory response after surgery, reducing muscle spasm, and promoting knee function recovery; thereby accelerating postoperative rehabilitation and the ability of patients to return to routine activities. To the best of our knowledge, cold dressing, cold packs, crushed ice bags and cooling pads have been used as common and traditional methods. With the development of cryotherapy techniques, CCD could simultaneously provide cold and compression to the knee surgery area; and this has become a routine in postoperative cryogenic nursing. Novel devices that generally comprise of specific cuffs, tubes and coolers make the operation simpler, maintain low temperatures for longer periods, and are more appropriate to the operation area. Compared with traditional cryotherapy, CCD brings not only pressurized therapy, but also extra cost. Therefore, better effectiveness for postoperative management may become the crucial factor for its application. For CCD, it remains undetermined whether compression or cryotherapy could actually reduce pain and swelling (Martimbianco et al. 2014). However, the limited evidence currently available from randomized trials is insufficient to draw certain conclusions on the comparison of CC and CA, in terms of the effectiveness of pain and swelling.

This meta-analysis included ten RCTs. To summarize, our review of recent English and Chinese literatures revealed that CC may have better therapeutic effects than CA. Furthermore, our meta-analysis suggests that: (1) patients who underwent CC had a better analgesic effect than those who underwent CA at POD2 and POD3; (2) CC had a better effect on swelling at POD1 and POD2; (3) it is noteworthy to mention that there were no reported serious adverse events in all included studies. Taken together, these results suggest that the effectiveness of CC is better than that of CA for patients undergoing knee surgery at the early stage of rehabilitation.

No previous meta-analyses have considered the comparison of the effectiveness of CC and CA after knee surgery, but several prior analyses were conducted on the effectiveness of CA. The conclusion of the meta-analyses on cryotherapy after knee surgery was common and helpful for clinical practice. Raynor et al. conducted a meta-analysis and revealed that cryotherapy after ACL reconstruction has a statistically significant benefit in postoperative pain control, while no improvement in postoperative range of motion or drainage was found (Raynor et al. 2005). By analyzing ten trials, the study of Martimbianco et al. was found to reveal that the use of CCD produced a significant reduction in pain scores at POD2 after arthroscopic ACL surgery compared to that without cryotherapy (Martimbianco et al. 2014). In brief, cryotherapy is effective for pain relief and swelling-control in patients undergoing knee surgery, especially at the early postoperative stage. In terms of our analysis results, CC was more effective than CA for pain remission at POD2 and POD3. Additionally, we found that CC had more advantages for decreasing swelling at POD1 and POD2. Based on cryotherapy, compression played an important role in pain relief and swelling-control. Theoretically, the persistence of vasoconstriction may be the main cause for coping with soft tissue injuries, through modulating swelling, pain, inflammation, metabolism, muscle spasm, and bleeding (Bleakley et al. 2004; Schaser et al. 2007). In practice, the dressing of low temperatures on the skin surface effectively enhances the healing of soft tissue injuries (Mejia et al. 2015). Therefore, the application of lower temperatures can reduce the need for pain medications and promote recovery (Trobec et al. 2008). In addition, Adie et al. consider that a low-temperature state might be able to reduce swelling by decreasing postoperative blood loss (Adie et al. 2012). However, the related molecular mechanism remains unclear. According to the conclusion of different meta-analyses, CA has been regarded as an effective pain relief and swelling-control nursing management for postoperative patients (Ni et al. 2015; Adie et al. 2010; Martimbianco et al. 2014). Furthermore, a systematic review on cryotherapy for acute soft tissue injury revealed the small but statistically significant effect of CC compared to CA (Bleakley et al. 2004). In concordance with other researchers’ studies, our outcome indicated that compression combined with cryotherapy effectively enhanced the curative effect. Due to restraints in the quantity of included trials and patients, no adverse reactions were reported in any of the included trials. However, adverse reactions of cryotherapy such as frostbite, cutaneous necrosis and neuropathy should not be ignored (Khoshnevis et al. 2015). Interestingly, soft tissue damage due to compression could be reduced by lowering the temperature, although compression, and has a potential impact on skin perfusion. This result may be connected with pro-inflammatory cytokine accumulation (Lee et al. 2014). To date, only one patient in the ice pack group developed transient peroneal nerve palsy, because cryotherapy time lasted for nearly 40 min (Cohn et al. 1989). Since 30 min has been generally adopted for cryotherapy treatment, no other adverse reactions were found.

Although the level of evidence was relatively low, this evidence still provided partial answers to the core questions raised in our study. In most included trials, the evaluation of outcomes was limited to a short observation period, which was between POD1 and POD3. Few English studies have evaluated the results of postoperative intervention. Therefore, it is necessary to perform further analyses that would include more sufficient long-term outcomes.

All studies that were included in this review had high risk of bias, recruited a small number of patients, and provided sparse data on most of our pre-established outcomes of interest; thereby precluding the pooling of their results into these meta-analyses. Furthermore, these studies were heterogenous in several aspects: these trials applied different forms of knee surgery (TKA, knee arthroplasty, ACL reconstruction and arthroscopic therapy), CA (ice bag and ice pack), CC (equal to CCD including Cryo Cuff system, Game ready coolsystems and Ever-cryo system), different frequencies and durations during sessions, and different follow-up periods. Inevitably, ice bag, cold pack and CCD also differ in handling, effect and efficiency. The main methodological limitations of these included studies were the lack of description of allocation concealment, difficulties in the blinding of participants, and outcome assessors; which were due to the nature of the intervention. This may in part be explained by the fact that old English and Chinese trials did not apply the standard recommendations for reporting clinical trials. Further studies with more consistent cryotherapy measurements and more standard data records would help to more accurately confirm this conclusion.

## Conclusion

CC and CA are both safe management methods for patients undergoing knee surgery. There is a moderate quality of evidence that CC is more effective in reducing pain at POD2 and POD3, coping with swelling at POD1 and POD2 after knee surgery. For patients who can afford CCD, we thought that these could obtain more benefits by applying CC at POD 1–3 after knee surgery. After the early stage, patients can have a choice (CC or CCD) for the remaining rehabilitation stages. The limited evidence currently available is insufficient to draw definitive conclusions on the effectiveness of this intervention for other outcomes such as the consumption of postoperative analgesic medications, knee range of motion, blood loss, hospital stay duration, quality of life measures and patient satisfaction. Moreover, well-designed, high quality randomized trials are needed to answer unsolved questions related to this comparison, as well as to supply more evidence-based conclusions and suggestions.

## Relevance to clinical practice

This review has provided an important contribution in selecting the optimum method for cryotherapy following knee surgery, by conducting a comparative evaluation between compressive cryotherapy and cryotherapy alone. More research is needed in this area to gain sufficient knowledge on other outcomes such as the consumption of postoperative analgesic medications, knee range of motion, blood loss, hospital stay duration, quality of life measures and patient satisfaction; since the current evidence obtained for this is weak. Additionally, more well-designed, high quality randomized trials are also expected.

## Abbreviations

TKA: total knee arthroplasty
ACL: anterior cruciate ligament
CCD: cold compression devices
CC: compressive cryotherapy
CA: cryotherapy alone
VAS: visual analog scale
POD: postoperative day
POW: postoperative week
MD: mean difference
SMD: standardized mean difference
